# Observation of Metal Nanoparticles for Acoustic Manipulation

**DOI:** 10.1002/advs.201600447

**Published:** 2017-02-14

**Authors:** Mian Chen, Feiyan Cai, Chen Wang, Zhiyong Wang, Long Meng, Fei Li, Pengfei Zhang, Xin Liu, Hairong Zheng

**Affiliations:** ^1^ Paul C. Lauterbur Research Center for Biomedical ImagingInstitute of Biomedical and Health EngineeringShenzhen Institutes of Advanced TechnologyChinese Academy of SciencesShenzhen518055China

**Keywords:** acoustic manipulation, hollow nanostructures, metal nanoparticles

## Abstract

Use of acoustic trapping for the manipulation of objects is invaluable to many applications from cellular subdivision to biological assays. Despite remarkable progress in a wide size range, the precise acoustic manipulation of 0D nanoparticles where all the structural dimensions are much smaller than the acoustic wavelength is still present challenges. This study reports on the observation of metal nanoparticles with different nanostructures for acoustic manipulation. Results for the first time exhibit that the hollow nanostructures play more important factor than size in the nanoscale acoustic manipulation. The acoustic levitation and swarm aggregations of the metal nanoparticles can be easily realized at low energy and clinically acceptable acoustic frequency by hollowing their nanostructures. In addition, the behaviors of swarm aggregations can be flexibly regulated by the applied voltage and frequency. This study anticipates that the strategy based on the unique properties of the metal hollow nanostructures and the manipulation method will be highly desirable for many applications.

## 1 Introduction

The capability to control and move nanoparticles on demand has attracted tremendous scientific interest and is of great technological significance for different application field such as bioengineering, chemical engineering, and pharmaceutical sciences.^1^, ^2^, ^3^, ^4^, ^5^, ^6^, ^7^ Up to now, optical tweezing has been exploited as one of the most powerful tools for nanoparticle manipulation and hence gains insight into biology phenomena and fundamental physics from molecular motor to protein folding.^8^, ^9^, ^10^, ^11^, ^12^, ^13^, ^14^ However, optical tweezing still faces the limitations of shallow penetration, low scale manipulation, complex devices and high laser power, which requires new efforts to seek other alternative technologies.

As a noninvasive and noncontact manipulation method, acoustic trapping has the advantage that it is able to simply levitate many kinds of objects from small living animal to bacteria.^15^, ^16^, ^17^, ^18^, ^19^, ^20^, ^21^ More importantly, the penetration of sound wave is much deeper than light in nontransparent media. In addition, acoustic trapping has been demonstrated the ability to restrict objects on the scale of large populations.^19^, ^20^ Techniques to achieve this goal are usually based on acoustic radiation force under a certain type of acoustic field, especially using counter‐propagating waves to set up standing wave nodes to trap the particles.^15^, ^17^, ^21^, ^22^, ^23^, ^24^ In general, the acoustic force on an object is proportional to the third power of the object radius in a standing wave field.^23^, ^24^ When the particle size reduces to nanoscale, the acoustic force decreases rapidly, and Brownian motion of the particles by the collisions with water molecules increases significantly. Thus, trapping nanoparticles requires much higher frequency and acoustic intensity. However, acoustic probes with high frequency such as hundreds of MHz or even more frequencies are difficult to implement. Meanwhile, the energy of high‐frequency waves is found to be obviously attenuation in the transmission.^25^ In addition, acoustic streaming could be induced by high acoustic intensities, resulting in instability of the trapping. All these factors have made the acoustic manipulation of nanoparticles where all the structural dimensions are much smaller than the acoustic wavelength to be extremely challenging. To the best of our knowledge, very little experimental verification of nanoparticle manipulation using acoustic field has been reported.

Metal nanoparticles (such as gold and silver) show many interesting optical, electronic, and chemical properties, which can be used in a range of applications involving photography, catalysis, biological sensors, and drug delivery.^26^, ^27^, ^28^, ^29^, ^30^, ^31^ For example, metal nanoparticles can be used as structural building blocks to construct more complex objects in a bottom‐up fashion, holding promise for nanoelectronics and nanorobotics applications.^32^, ^33^ In addition, gold or silver nanoparticle aggregate system has been proved to be an excellent substrate for surface‐enhanced Raman scattering applications, which provides the ability to significant amplify the Raman signal of nearby target molecules.^34^, ^35^ More interestingly, with the development of metal nanoparticle‐based drug delivery systems or mediated hyperthermia, the capability of acoustic manipulation to concentrate metal nanoparticle will offer a completely fresh approach to the treatment of many diseases including cancer. Thus, the performance of the acoustic manipulation of metal nanoparticles will offer more considerable promise as intelligent nanodevices. However, the intrinsic properties of most metal nanoparticles are significantly affected by its size, which means that it is not flexible enough for the acoustic manipulation by increasing the particle size.

In an attempt to solve this problem, we establish correspondence by looking at the relationship of acoustic‐stimulus response behavior between different nanostructures, including three different sizes of monodisperse silver nanocubes with solid inner, and their hollow nanostructures with various porosities. Results for the first time exhibit that the hollow nanostructures play more important factor than size in the nanoscale acoustic manipulation. The acoustic levitation and swarm aggregations of the metal nanoparticles can be easily realized at low energy and clinically acceptable acoustic frequency by hollowing their nanostructures. Meanwhile, the scale of the swarm aggregation can be easily controlled by switching the applied voltage. More interestingly, a controllable and reversible movement of the swarm aggregation in a certain range of distance is realized by adjusting the applied acoustic frequency. With the growing use of metal hollow nanostructures for biosensor and nanomedicine, acoustic manipulation with the unique properties mentioned above will be highly desirable for many applications.

## 2 Results

### 2.1 Fabrication of Metal Nanoparticles with Different Nanostructures

**Figure 1:**
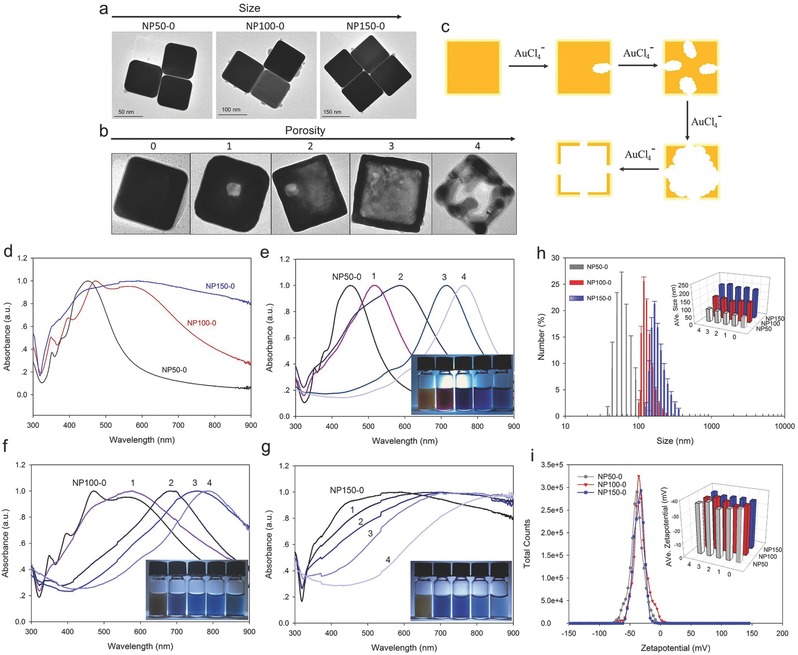
Characterization of the metal nanoparticles with different sizes and porosities. a) Transmission electron microscopy (TEM) images of three different sizes of metal nanoparticles with typical structures (silver nanocube with a solid inner). The edge lengths of them were about 50, 100, and 150 nm, namely NP50‐0, NP100‐0, and NP150‐0, respectively. b) TEM images of metal nanoparticles with the increasing porosities, which were synthesized by using the as‐prepared solid nanocubes as sacrificial templates, and then reacting with increasing amounts of HAuCl_4_ solution via the galvanic replacement reaction, as schematic illustrating in (c). The increasing porosity of the product was defined from 0 (no porosity) to 4 (the highest) status in each size level. d–g) Optical characterization of the products by UV–vis spectroscopy. The inserted pictures were the photograph of the as‐synthesized metal nanoparticles at various sizes: e) 50 nm; f) 100 nm; g) 150 nm and porosity (increasing from left to right). h) The size distribution and i) zeta potential of dispersed nanoparticles in aqueous were analyzed by dynamic light scattering and phase analysis light scattering, respectively. The inserted pictures showed the average size and the average zeta potential of the series of the products.

As one of the most representative and well‐established metal nanomaterials, silver nanocubes were chosen and synthesized according to the previous reports with some modifications.^36^, ^37^, ^38^ As shown by the transmission electron microscopy (TEM) images in **Figure** **1**a, highly monodisperse silver nanocubes with typical structures of about 50, 100, and 150 nm in edge length were synthesized, namely NP50‐0, NP100‐0, and NP150‐0, respectively. Optical characterization of the samples by UV–vis spectroscopy (Figure 1d) showed that the major localized surface plasmon resonance (LSPR) absorption band is redshifted from 450 to 590 nm with the increase of particle size, in agreement with those previously reports.^38^ To get the hollow nanostructures with various porosities, as‐prepared silver nanocubes were used as sacrificial templates and reacted with increasing amounts of chloroauric acid via the galvanic replacement reaction (Figure 1c). Figure 1b,e–g shows that greater extent of hollow interior was generated and the LSPR absorption peaks of the products (namely NP50‐1, NP50‐2, NP50‐3, and NP50‐4 with the increasing porosities from NP50‐0, and the same naming scheme for the products of NP100‐0 and NP150‐0, respectively) were redshifted, corresponding to a series of color changes. The LSPR band of NP150‐0 with larger size was relatively broad and thus less distinguishable than its products with the increasing porosities. The size distribution and zeta potential of dispersed nanoparticles in aqueous solution, which is essential to ensure that the results are reproducible, were analyzed by dynamic light scattering (DLS) and phase analysis light scattering, respectively. Figure 1h shows that the average hydrodynamic size of NP50‐0, NP100‐0, and NP150‐0 characterized by DLS was about 58.7, 118.1, and 165.8 nm, respectively. In general, the measurement result of particle size in solution by DLS is larger than that of by TEM. Figure 1i shows that the zeta potential of these nanoparticles was little different, according to a negative value of about −35 mV. After hollowing the structure of solid inner, there had little influence on the size distribution and zeta potential.

### 2.2 Acoustic Manipulation

**Figure 2:**
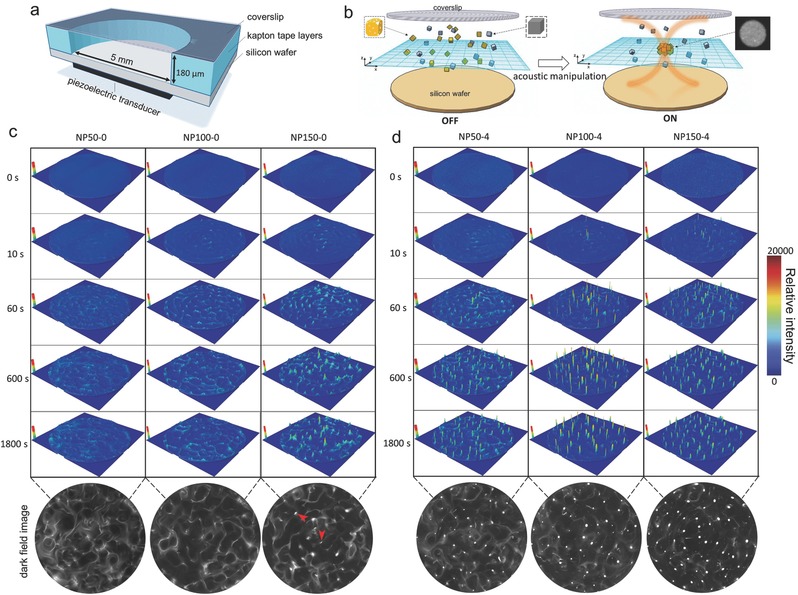
The acoustic manipulation behavior of metal nanoparticles between solid inner and the hollow nanostructures. a) Schematic illustration of acoustic manipulation system. The piezoelectric transducer was attached to the bottom center of a silicon wafer, with a sample reservoir mounted on top made of Kapton tape layers. A coverslip was used as an acoustic reflector on top. Experiments were carried out in continue sine waves with a resonant frequency of 4.5 MHz and the driving voltage of the acoustic field was 10 V_pp_. b) Schematic illustration of the acoustic manipulation effect between solid inner and the hollow nanostructures. The acoustic‐stimulus response behavior of c) the solid inner nanoparticles and d) the hollow nanoparticles. The images versus the levitation plane were recorded by dark‐field optical microscopy and also shown with surface plot by using the 3D surface plot plugin. In all scanned images, the size was 5 × 5 mm.

The acoustic levitation and aggregation of particles in water using a standing wave field provided a convenient way to study the influence of acoustic radiation force on different nanostructures. The acoustic manipulation system was assembled using an acoustic chamber as illustrated in **Figure** **2**a. The cylindrical acoustic chamber was designed to have a height of 180 µm and a diameter of 5 mm. Experiments were carried out in continue sine waves with a resonant frequency of 4.5 MHz. As described by formula λ = *cf* ^−1^, where *c* is the speed of sound in the medium (deionized water, 25 °C, *c* = 1496 m s^−1^) and *f* is the resonance frequency (*f* = 4.5 MHz), the sound wavelength λ was calculated to be 332 µm. Thus, there existed only one standing wave node plane that the time‐averaged force potential of the acoustic field was minimum in the vertical direction of the chamber. The driving voltage of the acoustic field was 10 V_pp_ (voltage peak to peak, an output power of 23.97 dBm, or 249.5 mW). Observation was carried out from above the chamber through the transparent glass reflector by dark‐field optical microscopy and was recovered at 0, 10, 60, 600, and 1800 s after acoustic manipulation, respectively. As shown in Video S1 (Supporting Information), in the absence of the acoustic field, the solid inner nanoparticles suspended in the cylindrical chamber showed typical Brownian motion, and there was no evidence of orderly migration, aggregation, or patterns (Figure 2c). When the acoustic field was switched on, it showed a time‐series properties of patterns on the bottom of the chamber with about 1 × 10^−9^ m solid inner nanoparticles (Figure 2c; Video S2, Supporting Information). The speed of the pattern formation was proportional to the size of the particles and the initial formation could be found within 10 s. Such phenomena are generally considered to be due to the inhomogeneous acoustic energy distribution in the acoustic fields. Meanwhile, the streaming eddies were set‐up during the pattern formation, which exerted drag and shear on the nanoparticles and hence influenced particles behavior in suspension. However, it was hard to predict or control the shapes of these patterns. In addition, it was worth noting that there was no evidence of acoustic levitation and aggregation of NP50‐0 and NP100‐0 in either the levitation plane or the vertical direction within 30 min. The reason was that there was deficiency of the acoustic pressure gradient to resist the influence of the gravitational force, diffusion, and the forces generated by acoustic streaming. When the particle size increased to 150 nm, the aggregation in the levitation plane was found after 60 s but for only two locations within 30 min, which was possibly a consequence of some small shape asymmetry (Figure 2c; Video  S2, Supporting Information).

**Figure 3:**
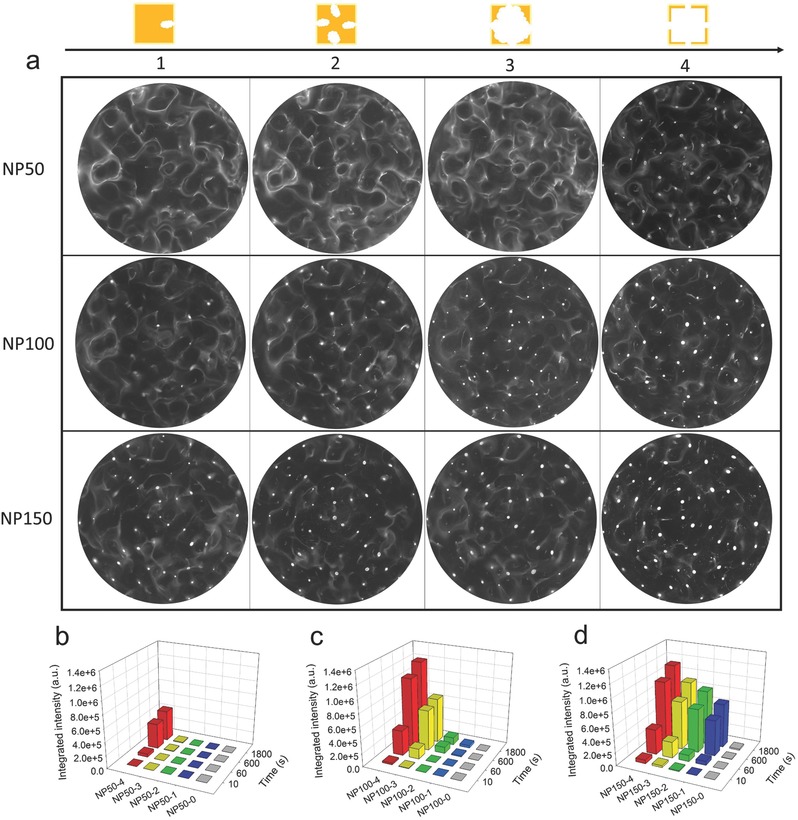
The acoustic levitation and aggregation among the hollow nanoparticles with various porosities. a) The acoustic‐stimulus response behavior of NP50, NP100, and NP150 with various porosities, respectively. Observation versus the levitation plane was carried out by dark‐field optical image and was recovered at 30 min after acoustic manipulation. The integrated intensity of the swarm aggregations of b) NP50, c) NP100, and d) NP150 in the whole chamber was calculated, respectively. The initial average intensity of each group (before acoustic manipulation) was normalized to 1.

Comparing to the nanostructures with solid inner, the acoustic‐stimulus response behavior of the hollow nanostructures showed some interesting differences under the same conditions, as illustrated in Figure 2b. Observation showed that NP50‐4, NP100‐4, and NP150‐4 with hollow nanostructures had a fast upward motion and then actually trapped in suspension about 90 ± 10 µm from the bottom of the chamber, corresponding to the nodes of the standing wave field in theory (Figure 2d; Video S3, Supporting Information). To further investigate the aggregation effect among the hollow nanostructures with various porosities, we calculated the integrated intensity of the swarm aggregations in the whole chamber. As shown in **Figure** **3** and Figures S1 and S2 (Supporting Information), it is interesting to note that the porosities are essential to the results of the time‐series properties of the swarm aggregations. Among the nanostructures, it was the easiest to trap NP150‐4, which had the biggest size and porosity. As shown in Figure 3d, for 10 s acoustic manipulation, the integrated intensity of the aggregation of NP150‐4 in the levitation plane was approximately 2.0 or 4.5 times as much as that of NP150‐3 or NP150‐2, respectively. Meanwhile, there was no evidence of aggregation of NP150‐1 and NP150‐0 in the same conditions. With the extension of the acoustic manipulation time to 30 min, the aggregation of NP150 with various porosities was less different to each other, but more obvious than that of NP150‐0 with solid inner. The trend from the experiments of NP100 was generally consistent with that of NP150, but the strength of the aggregation behavior was attenuation with the decrease of porosity (Figure 3a,c). For example, for 30 min acoustic manipulation, the integrated intensity of the aggregation of NP100‐4 in the levitation plane was approximately 1.8, 11.0, or 31.7 times as much as that of NP100‐3, NP100‐2, or NP100‐1, respectively. Meanwhile, there was no evidence of the aggregation of NP50 with the decrease of porosity from NP50‐4 (Figure 3b). According to the theory of the acoustic radiation force on solid spheres that it is proportional to the third power of the particle radius in standing waves, the acoustic manipulation of NP100 or NP50 would be more difficult than NP150. However, it was worth noting that the acoustic manipulation was operated at relatively low power, which did not undergo with optimal operating range for the extremely small size or porosity. Although we do not yet understand the details of the mechanism, the fact of that metal nanoparticles with hollow structures is better than that of solid inner for the acoustic manipulation has been shown for the first time.

### 2.3 Experiments by Varying the Driving Voltage

**Figure 4:**
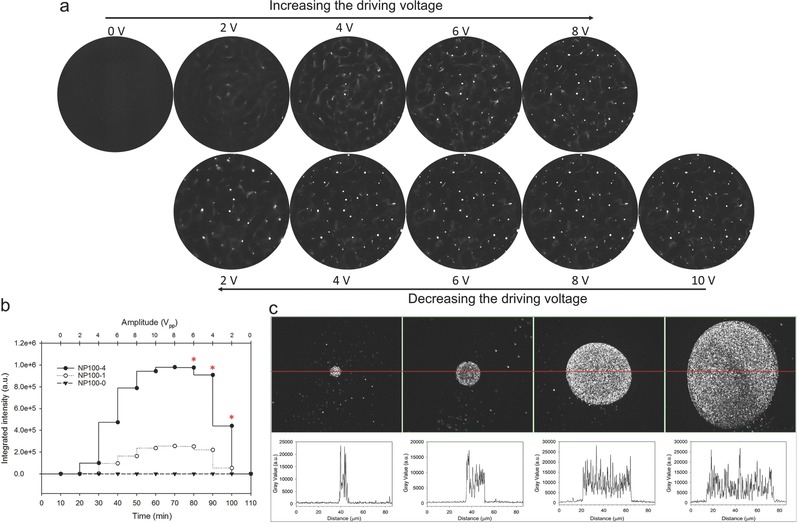
Experiments by varying the driving voltage. a) The acoustic manipulation of NP100‐4 by varying the driving voltage. The amplitude of the driving voltage was increased by 2 V_pp_ every 10 min from 0 to 10 V_pp_, followed by decrease from 10 to 0 V_pp_ with same rate. b) The amplitude dependence of the integrated intensity of the swarm aggregations in the whole chamber. c) The size regulation of the swarm aggregation by switching the applied voltage of 10 V_pp_ (for growth) and 4 V_pp_ (for maintain).

As noted above, the driving voltage is an important parameter for nanoparticles manipulation. Thus, the acoustic manipulation of NP100‐4 by varying the voltage was further investigated. Starting at the beginning, the amplitude of the driving voltage was increased by 2 V_pp_ every 10 min from 0 to 10 V_pp_. As shown in **Figure** **4**, the minimum driving voltage for the acoustic trapping of NP100‐4 was 4 V_pp_. The integrated intensity of the aggregation of NP100‐4 significantly increased with the further amplification of driving voltage. For the nonuniform acoustic energy distribution, the particles migration toward note showed accelerated motion. The average speeds (speed of NP100‐4 migration toward same nodes) of about 7.4, 30.2, 42.3 and 50.7 µm s^−1^ were observed upon increasing the applied voltage to 4, 6, 8, and 10 V_pp_, respectively. Meanwhile, the streaming eddies were also set up during the process. The average speeds (speed of NP100‐4 migration in the streaming eddies) of about 0.5, 3.2, 4.6, and 9.0 µm s^−1^ were observed, corresponding to the applied voltage of 4, 6, 8, and 10 V_pp_, respectively. The results indicated that the radiation force was more dominant for hollow nanoparticle manipulation than that of the streaming drag force at low power conditions. Meanwhile, it is beneficial for cases where the amplitude is sought at minimal applied voltage, which is essential to avoid unnecessary temperature increase and then depolarization of the transducers.

When the aggregations were established to a steady state with 10 V_pp_, the amplitude of the driving voltage was then decreased by 2 V_pp_ every 10 min from 10 to 0 V_pp_. It was worth noting that the aggregations of NP100‐4 trapping at the nodes had no decrease or disassembly with the reduction of the driving voltage to 6 V_pp_ (Figure 4a). In addition, it was still maintained over 90% and 40% of the aggregation of NP100‐4 at 4 V_pp_ and 2 V_pp_, respectively (Figure 4b). The results can be due to the secondary radiation force arising from the acoustic particle–particle interaction, which is proportional to the inverse of the squared separation distance. When the aggregation is established, the secondary radiation force produces a significant attraction, and thus the aggregation of nanoparticles is easier to maintain by the net effect of the primary and secondary acoustic radiation forces. Such property can be used as “pause button,” which is extremely useful for the regulation of acoustic trapping (Video S4, Supporting Information). As shown in Figure 4c, the scale of the swarm aggregation of NP100‐4 could display from several micrometers to tens of micrometers by switching the applied voltage of 10 V_pp_ (for growth) and 4 V_pp_ (for maintenance). Upon removal of the acoustic field, the swarm aggregation could not be maintained and fell down to the bottom. As demonstrated in Video S5 (Supporting Information), such aggregation and disaggregation could be easily repeated by switching ultrasound stimuli on and off.

### 2.4 Experiments by Varying the Applied Acoustic Frequency

**Figure 5:**
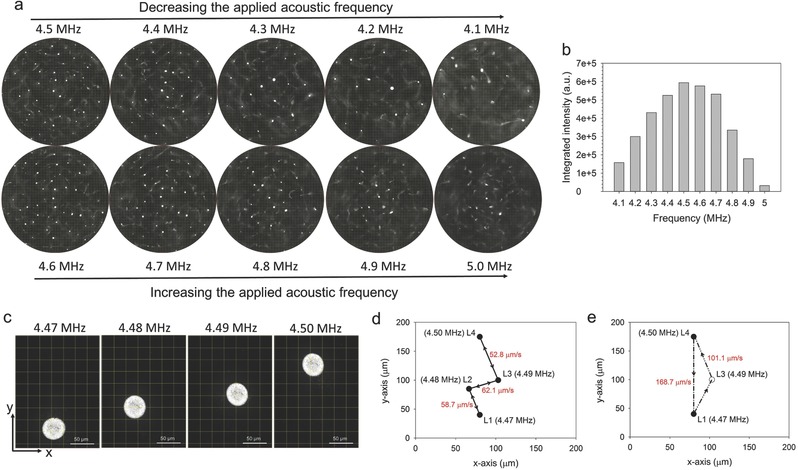
Experiments by varying the applied acoustic frequency. a) The aggregations of NP100‐4 in the levitation plane were observed in the frequency range from 4.1 to 5.0 MHz at 10 V_pp_ driving voltage. b) The integrated intensity of the swarm aggregations in the whole chamber, corresponding to the results of (a). c) Zoomed in optical micrograph and the location of the aggregation was firmly corresponded to the applied acoustic frequency from 4.47 to 4.50 MHz. The scale bar was 50 µm. d,e) Digitization of the orbiting motion and the location of the pressure nodes as the changes of the applied acoustic frequency from 4.47 to 4.50 MHz, according to Video S6 (Supporting Information). Red data denoted the average of the aggregation migration among the locations.

In addition to the driving voltage, the applied acoustic frequency is another important parameter for the acoustic manipulation. **Figure** **5**a shows that the aggregation of NP100‐4 in the levitation plane could be observed in the frequency range from 4.1 to 5.0 MHz at 10 V_pp_ driving voltage. The integrated intensity of the aggregations decreased with frequencies below or beyond 4.5 MHz (Figure 5b), corresponding to the power density of the piezoelectric transducer. Meanwhile, the location of the aggregations was changed by varying the applied acoustic frequency. This phenomenon was due to the changes in the location of the pressure nodes as the changes of the applied acoustic frequency, resulting in migration of the swarm toward the new location. As illustrated in Figure 5c,d and Video S6 (Supporting Information), the location of this aggregation was firmly corresponded to the applied acoustic frequency, such as L1 (location 1) for 4.47 MHz, L2 for 4.48 MHz, L3 for 4.49 MHz, and L4 for 4.50 MHz, respectively. Thus, a controllable and reversible movement of the swarm aggregation in a certain range of distance can be realized by adjusting the applied acoustic frequency. The average speeds (speed of the aggregation migration among the locations) of about 58.7, 62.1, and 52.8 µm s^−1^ were observed upon increasing the applied acoustic frequency by every 0.01 MHz from 4.47 to 4.50 MHz, respectively. Such movement orbit and the average speeds were little difference with the reverse operation from 4.50 to 4.47 MHz by every 0.01 MHz (Figure 5d). It was worth to note that there existed some differences when directly switched the applied acoustic frequency between 4.47 and 4.50 MHz. As shown in Figure 5e and Video S6 (Supporting Information), the movement orbit (L4 → L1) was straight and the average speed was increased to 168.7 µm s^−1^ with the operation direction from 4.50 to 4.47 MHz. Interestingly, with the opposite operation, the movement orbit (L1 → L3 → L4) was drifted from the original route and the average speed was changed to 101.1 µm s^−1^. These phenomena are expected as the changes of acoustic field, but the detail mechanism is still difficult to explain. In the system, the shift distance and the average speed of the swarm aggregation could reach up to 250 µm and 494.8 µm s^−1^, respectively.

## 3 Discussion

Up to now, only several observed evidences of acoustic levitation and aggregation of nanoparticles have been reported yet. For example, it has been reported that 5 nm diamond nanoparticles could be manipulated into nodal patterns using acoustic standing waves field.^24^ However, driving by the high surface energy, the diamond nanoparticles with ultra‐small size are particularly known to form large agglomerates.^39^, ^40^, ^41^ As the nanoparticles in the experiments were not treated with a surfactant to prevent the self‐assembly, the actual manipulation effect may depend on the size of agglomerates rather than that of single nanoparticle. Recently, different metal nanorods have also been explored for acoustic manipulation, exhibiting interesting behaviors in ultrasonic response, including levitation, chain assembly, reversible swarming, axial propulsion, rolling, ring patterns, streak patterns, and the others.^42^, ^43^, ^44^ Although the nanorods in these experiments showed nanosized structures on tangent plane, the dominated parameter for the nanoscale acoustic manipulation should be further discussed to exclude the influence of their microscale length. In other words, the obtained experimental results and predictions may not agree well for the acoustic manipulation of zero‐dimensional nanoparticles where all the structural dimensions are much smaller than the acoustic wavelength.

In this study, the highly monodisperse and structure‐tunable silver nanocubes were investigated (Figure 1). Meanwhile, acoustic levitation and aggregation events using a standing wave field provide a convenient way to study the dominated parameter of acoustic manipulation in nanoscale. The observed events showed that it was difficult to acoustic levitation or aggregation the solid inner nanoparticles with size from 50 to 150 nm (Figure 2c). The results indicate that the size parameter is not important for acoustic manipulation when the particle size is much smaller than the acoustic wavelength. According to the previous theory,^23^, ^24^, ^45^, ^46^, ^47^, ^48^ the primary acoustic radiation force *F* on small particles can be calculated as Equation (1)

(1) $$F_{\max}=\frac{\pi P^{2}V_{P}}{2\lambda }\frac{1}{\rho_{M}C_{M}^{2}}\left(\frac{5\rho_{P}-2\rho_{M}}{2\rho_{P}+\rho_{M}}-\frac{\rho_{M}C_{M}^{2}}{\rho_{P}C_{P}^{2}}\right)$$

where *P* is acoustic pressure, *V*_P_ is the volume of metal particle, ρ_P_ and ρ_M_ are the density of metal particle and medium, *C*_P_ and *C*_M_ are the speed of sound in particle and medium, respectively. Details are listed in Table S1 (Supporting Information). Ignoring the impact of the surfactant and other uncertainties, the max primary acoustic radiation force, gravitational force, and buoyancy for 100 nm silver nanocube can be calculated to be about 4 × 10^−17^, 10 × 10^−17^, and 1 × 10^−17^ N, respectively. The Brownian motion is an important topic when manipulate nanoparticles. However, the active Brownian motion of a nanoparticle collided with water molecules can be interpreted as a random walk. Meanwhile, the drag force and the shear stress in the streaming eddies do not contribute to nanoparticle levitation in our system. Thus, there is no enough force to drive the nanoparticles into the levitation plane.

In contrast, the time‐series properties of acoustic levitation and swarm aggregations can be realized by hollowing the silver nanocubes in the same conditions (Figure 2d). With the increase of the porosities, the acoustic levitation and swarm aggregations are more significant (Figure 3). These data provide an opportunity to define hollowing structure as more important factor than size in nanoscale acoustic manipulation, to which little consideration has been given yet. One of the possible reasons is that the gravity acting on the nanoparticle is weakened by the hollowing nanostructures. Meanwhile, the density of the alloy frame (Ag/Au) is still higher than that of the surrounding fluid. In addition, the hollow nanostructures have larger compressibility than solid nanostructures. Thus, the primary acoustic radiation force will be enhanced and easier to drive the metal hollow nanostructures into the levitation plane. Due to the alloy frame density and its compressibility,^23^, ^49^ we also found that the secondary forces arising from the acoustic particle to particle interaction became much more significant, as shown in Video S7 (Supporting Information). The net effect of the primary and secondary acoustic radiation forces makes the acoustic levitation and swarm aggregations of the metal hollow nanostructures to be easily realized at low energy and clinically acceptable acoustic frequency.

The ability to flexibly regulate the behaviors of swarm aggregation is important for acoustic manipulation. In this system, we can regulate the speed of the hollow nanostructures migration toward aggregation nodes by varying the driving voltage. In addition, the scale of the swarm aggregation could grow from several micrometers to tens of micrometers. More interestingly, when the aggregation is established, the aggregation of the hollow nanostructures is easier to maintain with only one‐sixth of the output power (Figure 4). The acoustic manipulation operated at relatively low power is not only beneficial to avoid acoustic streaming, but also essential to avoid unnecessary temperature increase and then depolarization of the transducers. In addition to the regulation fashion of voltage, the swarm aggregation of the hollow nanostructures can be moved in a controlled manner by varying the applied acoustic frequency (Figure 5). Within a certain range, the location of the swarm aggregation is firmly corresponded to the applied acoustic frequency, but it showed some interesting differences in the movement speed and orbit by the changes of frequency in magnitude and direction.

Finally, metal hollow nanoparticles with unique properties have attracted tremendous scientific interest, especially in drug delivery and cancer therapy.^27^, ^29^, ^50^, ^51^, ^52^, ^53^ Owing to their nanometer size, they exhibit large surface‐to‐volume ratios that can improve drug loading efficiency, cellular internalization, and systemic delivery with low immunogenicity. After surface functionalization, these nanoparticles can further be used as stimuli‐responsive nanocarriers for drug delivery. On the other hand, most of metal hollow nanoparticles have a high efficiency for the conversion of light energy into heat, providing the photothermal therapy of cancer. Despite the concept being far from the clinical applications, the observation of metal hollow nanostructures for acoustic manipulation will offer considerable promise for a more precise and individualized treatment of cancer.

## 4 Conclusions

In summary, we have first time observed that the hollow nanostructures play more important factor than size in the nanoscale acoustic manipulation. The acoustic levitation and swarm aggregations of the metal nanoparticles can be easily realized at low energy and clinically acceptable acoustic frequency by hollowing their nanostructures. In addition, the behaviors of swarm aggregations can be flexibly regulated by the applied voltage and frequency. We anticipate that the strategy based on the unique properties of the metal hollow nanostructures and the manipulation method will be highly desirable for many applications.

## 5 Experimental Section

*Nanoparticles Preparation*: Silver solid nanocubes with three different sizes were prepared, according to the previous reports with some modifications.^36^, ^37^, ^38^ Briefly, 20 mL of ethylene glycol (EG, Aladdin) was added into a 100 mL glass vial and heated under magnetic stirring in an oil bath at 150 °C for 1 h. Then, 240 µL of NaSH (Aladdin, 3 × 10^−3^ m in EG) was quickly injected into the heated solution. Two minutes later, 2 mL of HCl (Sigma, 3 × 10^−3^ m in EG) was quickly added into the vial, followed by addition 5 mL of poly(vinyl pyrrolidone) (PVP, *M*_W_ ≈ 58 000, Aladdin, 20 mg mL^−1^ in EG). After another 2 min, pipetted 1.6 mL of silver trifluoroacetate (C_2_F_3_O_2_Ag, Aladdin, 282 × 10^−3^ m in EG) into the vial. When the solution had a desired optical extinction peak as confirmed by UV–vis spectroscopy, the reaction was quenched by placing the vial in an ice‐water bath. The final product was collected by centrifugation and then washed with acetone and nanopure water. The morphology of the products was examined by transmission electron microscopy (Tecnai G2 Spirit 120 kV). The hollow nanostructures with various porosities were synthesized by using silver nanocubes as sacrificial templates. Briefly, transferred 5 mL of PVP solution (1 mg mL^−1^ in water) with silver nanocubes into a 50 mL round bottom flask and heated under magnetic stirring in an oil bath at 90 °C for 10 min. Then, chloroauric acid (HAuCl_4_, Sigma, 0.5 × 10^−3^ m in water) was added to the flask at a slow rate until the solution had a desired optical extinction peak as confirmed by UV–vis spectroscopy. Finally, the hollow nanostructures were collected by centrifugation and then washed with saturated NaCl solution to remove AgCl and with water several times to remove PVP and NaCl.

*Acoustic Experiments*: The acoustic manipulation system was constructed as previously described with small modifications.^42^, ^43^, ^44^ Briefly, the cylindrical reservoir with a height of 150 µm and a circular hole of 5 mm diameter cut in the center was created by attaching three layers of polyimide Kapton tapes on a piece of polished silicon wafer (76.2 mm × 76.2 mm × 381 µm). A piezoelectric ceramic transducer (PZT) 4.5 MHz (Kunshan Risun Electronic Co., Ltd, China) was attached by conductive epoxy glue to the back of the silicon wafer to generate acoustic waves. The transducer was connected to a function generator that outputted sine waves (a Tektronix arbitrary/function generator AFG 3102 is used to generate the baseband transmitted signal). In a typical experiment, 10 µL of nanoparticle (1 × 10^−9^ m) in ultrapure water was added to the cylindrical reservoir. Then, the cylindrical reservoir was covered by a coverslip that was served as the sound reflector. A Leica DM4000M optical microscope and a commercial video capturing bundle (optiMOS sCMOS camera, QImaging) were used for observing the particles and recording videos. As the metal nanoparticles exhibit LSPRs and brilliant elastic light‐scattering properties, it is sufficient to detect individual nanoparticles in a dark‐field optical microscopy with high spatial resolution. Experiments were carried out in continue sine waves with a frequency between 4 and 5 MHz, and the highest output voltage of the acoustic field was 10 V_pp_. Images were observed and analyzed by using both the ImageJ software and Image‐Pro Premier software (Meyer Instruments, Inc.).

## Supporting information

As a service to our authors and readers, this journal provides supporting information supplied by the authors. Such materials are peer reviewed and may be re‐organized for online delivery, but are not copy‐edited or typeset. Technical support issues arising from supporting information (other than missing files) should be addressed to the authors.

Supplementary

Supplementary

Supplementary

Supplementary

Supplementary

Supplementary

Supplementary

Supplementary
