# Supplementary material for: Observation of Metal Nanoparticles for Acoustic Manipulation
Source: Adv Sci (Weinh). 2017 Feb 14;4(5):1600447. doi: 10.1002/advs.201600447 (PMC5441484; doi:10.1002/advs.201600447)
Supplement: Supplementary file 1 — Supplementary [file ADVS-4-na-s001.pdf]

Copyright WILEY-VCH Verlag GmbH & Co. KGaA, 69469 Weinheim, Germany, 2017.

ADVANCED SCIENCE
Open Access

Supporting Information

for *Adv. Sci.,* DOI: 10.1002/advs.201600447

Observation of Metal Nanoparticles for Acoustic Manipulation

*Mian Chen, Feiyan Cai, Chen Wang, Zhiyong Wang, Long Meng, Fei Li, Pengfei Zhang, Xin Liu, and Hairong Zheng**

Copyright WILEY-VCH Verlag GmbH & Co. KGaA, 69469 Weinheim, Germany, 2016.

Supporting Information

Observation of metal nanoparticles for acoustic manipulation

*Mian Chen, Feiyan Cai, Chen Wang, Zhiyong Wang, Long Meng, Fei Li, Pengfei Zhang, Xin Liu and Hairong Zheng**

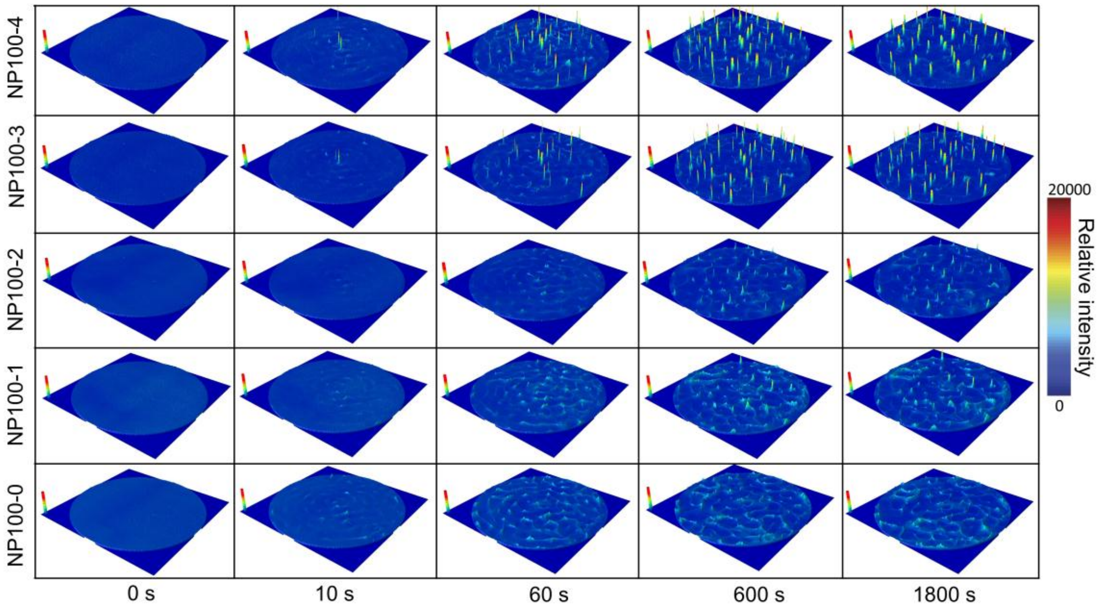

Figure S1**.** Acoustic manipulation of NP100 with various porosities. The images versus the levitation plane were recorded by dark-field optical microscopy and shown with surface plot by using the three-dimensional (3-D) Surface Plot plugin. In all scanned images the size is 5 × 5 mm.

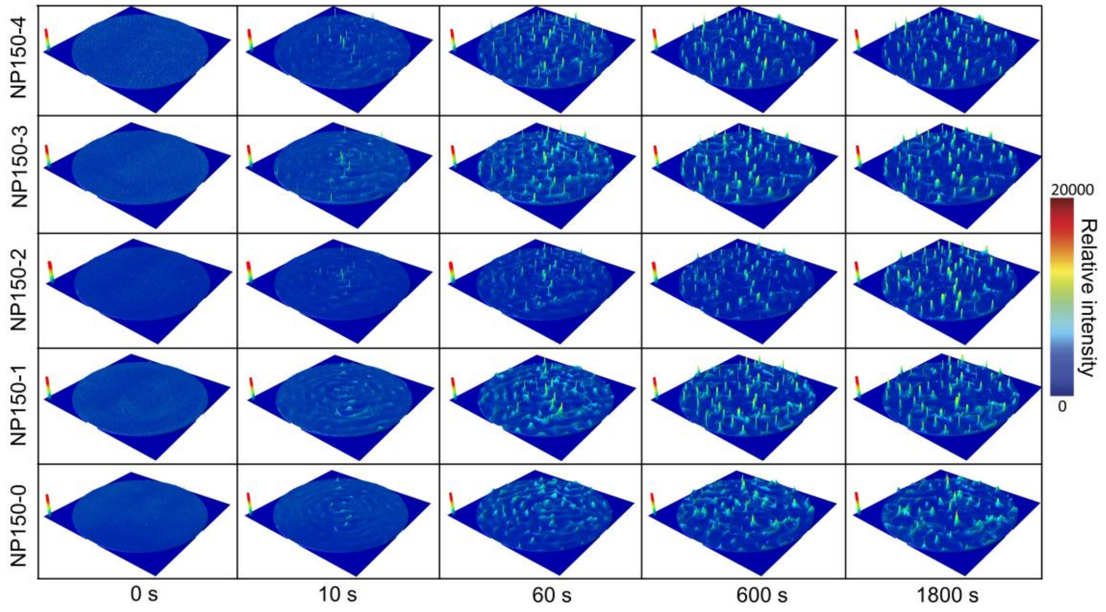

Figure S2. Acoustic manipulation of NP150 with various porosities. The images versus the levitation plane were recorded by dark-field optical microscopy and shown with surface plot by using the three-dimensional (3-D) Surface Plot plugin. In all scanned images the size is 5 × 5 mm.

Table S1. Notation and value

ρ_P	the density of particle<br>for pure metals Ag: 10.5 g·cm^{-3}; Au: 19.32 g·cm^{-3};
ρ_M	the density of medium<br>for pure water: 1 g·cm^{-3};
C_P	the speed of sound in particle<br>for pure metals Ag: 3650 m·s^{-1}; Au: 3240 m·s^{-1};
C_M	the speed of sound in medium<br>for pure water: 1496 m·s^{-1}
V_P	the volume of particle<br>1.0×10^{-21} m^3 for 100 nm nanocube
P	maximum acoustic pressure<br>about 1.0×10^4 Pa (measured at 4.5 MHz and 10 V_{pp}).
λ	the sound wavelength<br>about 332 μm (for 4.5 MHz in water)

Videos

1. Video S1

Brownian motion. In the absence of the acoustic field, 50 nm silver nanocubes suspended in the cylindrical chamber showed typical Brownian motion. The observations were conducted

under the upright microscope Leica DM4000M with a HC PL Fluotar 50×/0.55 BD objective. The movie was captured at 100 fps and was played at 100 fps.

2. Video S2

Solid nanostructures for acoustic manipulation. 50, 100 and 150 nm metal nanoparticles with solid nanostructures (about 1 nM) were exposed to the acoustic field (continue sine waves with a resonant frequency of 4.5 MHz and the driving voltage of 10 V_{PP}), respectively. For smaller solid nanoparticles (such as 50 or 100 nm), the acoustic streaming force is dominant over the radiation force, so there is no evidence of acoustic levitation and aggregation. The observations were conducted with an N PLAN EPI 5×/0.12 BD objective. The movie was captured at 5 fps and was played at 20 fps.

3. Video S3

Hollow nanostructures for acoustic manipulation. 100 nm metal nanoparticles with hollow nanostructures (NP100-4, about 1 nM) were exposed to the acoustic field (continue sine waves with a resonant frequency of 4.5 MHz and the driving voltage of 10 V_{PP}). It showed that the hollow nanostructures had a fast upward motion and then actually trapped in suspension, which indicated that the radiation force was dominant over the acoustic streaming force. The observations were conducted with a HCX PL FLUOTAR 10×/0.30 and an N PLAN EPI 5×/0.12 BD objective, respectively. The movie was captured at 5 fps and was played at 20 fps.

4. Video S4

Experiments by varying the driving voltage between 4 and 10 V_{pp}. When the aggregation is established, the secondary radiation force arising from the acoustic particle-particle interaction produces a significant attraction, and thus the aggregation of nanoparticles is easier to maintain by the net effect of the primary and secondary acoustic radiation forces. It showed that 4 V_{pp} and 10 V_{pp} could be used for maintaining and growing the aggregation, respectively.

The observations were conducted with a HCX PL FLUOTAR 10×/0.30 objective. The movie was captured at 5 fps and was played at 5 fps.

5. Video S5

Experiments by varying the driving voltage between 10 and 0 V_{pp}. The aggregation and dis-aggregation could be easily repeated by switching the driving voltage between 10 Vpp and 0 Vpp. The observations were conducted with a HCX PL FLUOTAR 10×/0.30 objective. The movie was captured at 5 fps and was played at 20 fps.

6. Video S6

Experiments by varying the applied acoustic frequency. The location of the swarm aggregation was firmly corresponded to the applied acoustic frequency, but it showed some interesting differences in the movement speed and orbit by the changes of frequency in magnitude and direction. The observations were conducted with a HCX PL FLUOTAR 20×/0.40 objective. The movie was captured at 5 fps and was played at 5 fps.

7. Video S7

The effect of the secondary forces in the aggregation process. The observations were conducted with a HCX PL FLUOTAR 20×/0.40 objective. The movie was captured at 5 fps and was played at 20 fps.
